# Compressive femoral neuropathy caused by anticoagulant therapy induced retroperitoneal hematoma: A case report

**DOI:** 10.1097/MD.0000000000028876

**Published:** 2022-02-18

**Authors:** Tae-Hoon Kim, Da-Jung Lee, Wanil Kim, Hwan-Kwon Do

**Affiliations:** a Department of Physical Medicine and Rehabilitation, Haeundae Paik Hospital, Inje University College of Medicine, Busan, Republic of Korea; b Department of Biochemistry, Department of Convergence Medical Science, Institute of Health Sciences, Gyeongsang National University College of Medicine, Jinju, Republic of Korea.

**Keywords:** anticoagulant therapy, femoral neuropathy, retroperitoneal hematoma, vascular thrombosis

## Abstract

**Rationale:**

Spontaneous retroperitoneal hematomas due to anticoagulant therapy rarely occur. Retroperitoneal hematomas can cause severe pain in the groin, quadriceps femoris muscle weakness, hemodynamic instability, and abdominal distension. They rarely cause compressive neuropathy of the femoral nerve transversing the iliacus muscle. Differential diagnosis is not easy because they have similar clinical features to retroperitoneal hematomas.

**Patient concerns:**

A 72-year-old female patient whose right arm was stuck in a bookshelf for 5 days developed right cephalic vein thrombosis. After 5 days of intravenous heparin therapy for venous thrombosis, she presented with sudden right groin pain, right leg paresis, hemodynamic instability, and abdominal distension.

**Diagnosis:**

Emergency abdominal and pelvic CT showed a large number of hematomas in the bilateral retroperitoneal space with active bleeding of the right lumbar artery. An electrodiagnostic study was performed 2 weeks later to check for neuromuscular damage in the right lower extremity, and right compressive femoral neuropathy was confirmed.

**Interventions:**

Heparin therapy was discontinued; emergency embolization of the lumbar artery was performed. After 2 weeks, the patient started receiving physical, occupational, and transcutaneous electrical stimulation therapies.

**Outcomes:**

She became hemodynamically stable after arterial embolization; a significant decrease in hematoma and patency of the femoral nerve was confirmed on follow-up pelvic MRI. After 2 months of comprehensive rehabilitation, the muscle strength of the right leg significantly improved, and the pain disappeared.

**Lessons:**

Although rare, spontaneous retroperitoneal hematomas may occur in patients receiving anticoagulant medications. They may even occur in patients receiving emergency anticoagulant therapy. Compressive femoral neuropathy due to retroperitoneal hematomas should be considered if muscle weakness and groin pain are observed. Early diagnosis and appropriate treatment plan of compressive femoral neuropathy due to retroperitoneal hematoma are helpful for a good prognosis.

## 1 Introduction

Anticoagulant therapy for vascular thromboses such as venous thrombosis, pulmonary embolism, and coronary artery disease can cause various complications, including thrombocytopenia and bleeding.^[1]^ Spontaneous retroperitoneal hematoma, defined as bleeding into the retroperitoneal space without associated trauma or iatrogenic manipulation, is rare but may occur as a complication of anticoagulant therapy.^[^2^,^^3^^]^ Retroperitoneal hematoma can cause severe pain in the groin area, quadriceps femoris muscle weakness, hemodynamic instability, and abdominal distension.^[4]^ In addition, retroperitoneal hematoma can cause compressive neuropathy of the femoral nerve as the nerve passes through the closed fibrous compartment formed by the iliac fascia and ileum.^[5]^ In femoral neuropathy, weakness of iliopsoas and quadriceps femoris muscle occurs, accompanied by patellar hyporeflexia and hypoesthesia on the anterior segment of the leg.^[6]^ However, since the symptoms of femoral neuropathy are similar to those of retroperitoneal hematoma, it is difficult to differentiate the two based on clinical symptoms alone.

Femoral neuropathy due to retroperitoneal hematoma has been described infrequently and underreported because it is rare, and cases that occur during emergency trauma management have not been reported. This study reports the first case of spontaneous retroperitoneal hematoma comorbid with femoral neuropathy in a patient who underwent emergency anticoagulant therapy during upper arm trauma management.

## 2 Case report

### 2.1 Patient information

A 72-year-old female patient with no underlying disease was rescued after 5 days of having her right upper arm stuck between the bookshelf and the wall without water or food, then transferred to an emergency center. On admission, her vital signs were stable, but she was severely dehydrated and malnourished, and her right arm showed swelling and multiple abrasions of the medial side.

### 2.2 Clinical findings

She complained of hypoesthesia in the distal arm with grade 2 (poor) right finger flexion, elbow flexion, and extension on manual muscle testing (MMT), but both legs and left arm were confirmed as grade 5 (normal). The radial and ulnar arteries were patent on Doppler ultrasonography, but an angio-upper extremity computed tomography (CT) scan revealed thrombosis of the right cephalic vein. Intravenous heparin therapy was initiated, and hydration and nutritional therapy were started for accompanying acute kidney injury, rhabdomyolysis, and malnutrition.

**Figure 1:**
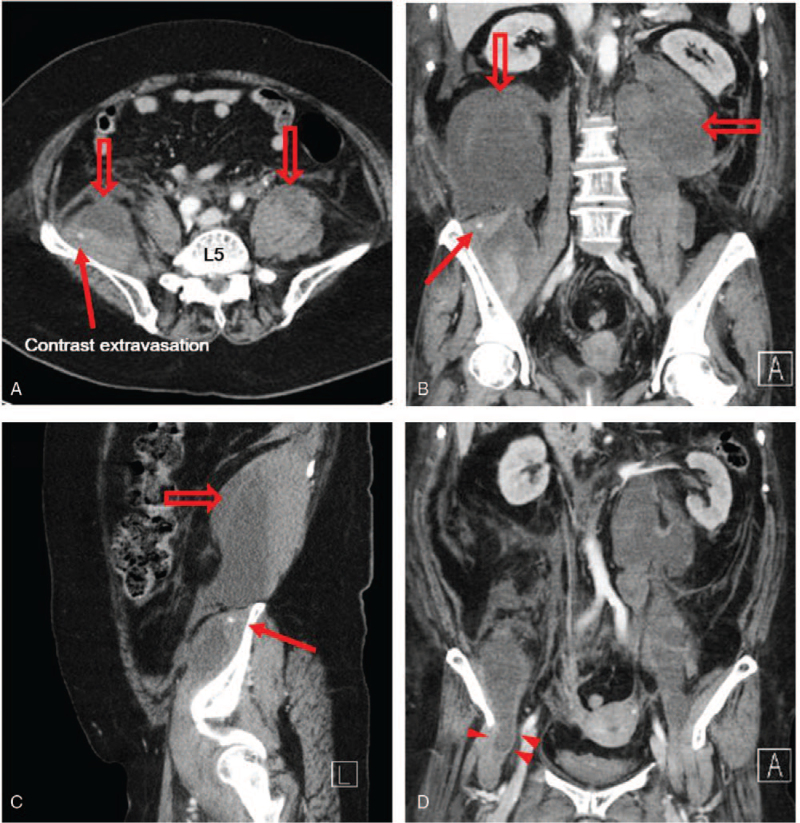
A large number of hematomas in the bilateral retroperitoneal space with active bleeding on abdominal and pelvic CT. (A) Axial image at the lumbar vertebra 5 level; (B) coronal image; (C) sagittal image. The hollow red arrows indicate the large number of bilateral retroperitoneal hematomas involving the psoas and iliacus muscles. The red arrows indicate the contrast extravasation of the right lumbar artery. The red arrowhead indicates the iliacus hematoma extending between the ileum and the inguinal ligament. L5 = 5th lumbar vertebra.

After 5 days, the patient suddenly complained of severe pain on the numeric rating scale (NRS) of 8 points and paralysis of the right leg. MMT grade 1 (trace) of the right quadriceps femoris and increased abdominal distension with dyspnea were observed on physical examination. Her blood pressure was low at 80 mm Hg of systolic blood pressure and 50 mm Hg of diastolic blood pressure, and hemoglobin dropped from 10.4 to 5.9 g/dL compared with initial laboratory studies. An emergency contrast-enhanced abdominal and pelvic CT was performed, and a large number of bilateral retroperitoneal hematomas, involving the iliopsoas muscle, with active bleeding in the right lumbar artery were confirmed (Fig. 1). Heparin therapy was immediately discontinued, and emergency embolization of the lumbar artery was performed. Although hemodynamically stable after the emergency embolization, she complained of pain in the right groin throughout the following week. On physical examination, hip flexion strength was MMT grade 2 (poor), knee extension was grade 1 (trace), deep tendon reflex of the knee had decreased compared to the left, and the anterior thigh sensation also reduced.

### 2.3 Diagnostic assessment

An electrodiagnostic study using Viking Select (Nicolet, San Carlos, CA) was performed 2 weeks after embolization to check for neuromuscular lesions.

A motor nerve conduction study (NCS) found no nerve conduction in the right femoral nerve. In addition, a sensory NCS also found no nerve conduction in the right saphenous nerve (Table 1). Needle electromyography (EMG) identified abnormal spontaneous activities in the right iliopsoas, rectus femoris, and vastus medialis muscles. Also, the recruitment of motor unit action potentials was significantly reduced or absent during muscle contraction. NCS and EMG results for the left lower extremity were normal ranged, and EMG for the right L4, L5, S1 paraspinal muscle was also normal (Table 2). After comprehensively evaluating the imaging study and electrodiagnostic findings on the paralysis of the right leg, the patient was diagnosed with right compressive femoral neuropathy in the iliacus muscle, caused by spontaneous retroperitoneal hematoma. The right arm paralysis was diagnosed with multiple mononeuropathy, including median, ulnar, radial, and musculocutaneous nerves, attributable to mechanical compression by the upper arm stuck. In addition, a vascular ultrasound for the right arm confirmed that there was no residual thrombosis after the discontinuation of intravenous heparin therapy.

**Table 1:** Results of nerve conduction study.

Nerve		Stimulation site	Latency (ms)	Amplitude (mV)	NCV (m/s)
Motor	Rt femoral (VM recording)	Below inguinal ligament	No potential	No potential	
		Above inguinal ligament	No potential	No potential	
	Lt femoral (VM recording)	Below inguinal ligament	2.7	2.1	
		Above inguinal ligament	6.6	2.3	51.1
Sensory	Rt saphenous	Calf	No potential	No potential	
	Lt saphenous	Calf	2.0	2.4	49.0

Lt = left, NCV = nerve conduction velocity, Rt = right, VM = vastus medialis.

**Table 2:** Results of needle electromyography.

		Denervation potentials			
Muscle	Insertional activity	Fibs	PSWs	Polyphasic MUAP	Interference pattern
Rt iliopsoas	Increased	1+	3+	Many	Marked reduced
Rt rectus femoris	Increased	2+	3+	–	None
Rt vastus medialis	Increased	2+	3+	–	None
Rt tibialis anterior	Normal	–	–		Full
Rt peroneus longus	Normal	–	–		Full
Rt L4 paraspinalis	Normal	–	–		
Rt L5 paraspinalis	Normal	–	–		
Rt S1 paraspinalis	Normal	–	–		

Fibs = fibrillation potentials, MUAP = motor unit action potentials, PSWs = positive sharp waves, Rt = right.

### 2.4 Therapeutic intervention

The patient received daily comprehensive physical therapies, including strengthening exercises for the right leg, standing and gait training with walking aid, and electrical stimulation therapy. In addition, occupational therapies were received to improve the muscle strength and function of the hand, while non-steroidal anti-inflammatory drugs and pregabalin were administered for pain control. The patient maintained stable vital signs without any complications during the rehabilitation treatment.

### 2.5 Follow-up and outcomes

**Figure 2:**
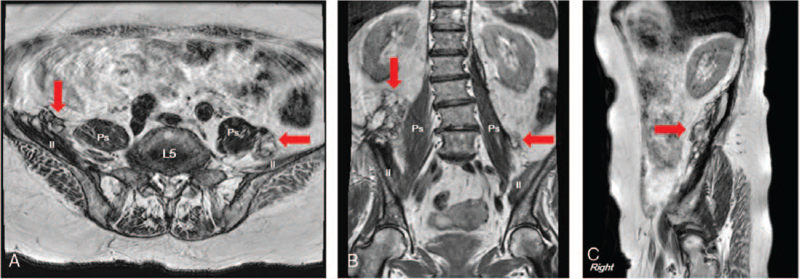
Pelvic magnetic resonance imaging at follow-up. (A) Axial plane at the lumbar vertebra 5 level; (B) coronal plane; (C) sagittal plane. The red arrows indicate a decrease in the extent of the previously noted liquefied hematoma in the right retroperitoneum and mostly resorbed hematoma in the left retroperitoneum. Il = iliacus muscle, L5 = 5th lumbar vertebra, Ps = psoas muscle.

After 2 months, physical examination confirmed significant improvement in the hip flexion to MMT grade 4 (good) and knee extension to grade 3 (fair), and hand function was restored enough to perform daily activities. There was mild hypoesthesia of the anterior thigh, but the patient no longer complained of pain. The follow-up pelvic magnetic resonance imaging (MRI) showed a significant decrease in hematomas and patency of the femoral nerve (Fig. 2).

## 3 Discussion

The femoral nerve, the largest branch in the lumbar plexus, is generated in the posterior division of ventral rami in the second to fourth lumbar nerves. It passes through the inside of the psoas muscle, and it is then runs between the psoas tendon below the iliacus fascia and the iliacus muscle to the femoral canal below the inguinal ligament.^[4]^ The femoral nerve is branched out to provide motor innervations to the quadriceps, sartorius, pectineus, and iliopsoas muscles, and the sensory innervations to the anteromedial thigh and the medial leg.^[2]^ Thus, when femoral nerve palsy occurs, it can cause weakness in hip flexion and knee extension and the loss of sensation to the area supplied by the femoral nerve. Femoral nerve palsy can result from various causes, which include direct injury to the nerve; a mass that blocks or traps the nerve; a long-term compression to the nerve; a catheter inserted into the femoral artery for surgery; pelvic fracture; radiation to the pelvis; and hemorrhage in the retroperitoneal space.^[5]^

Retroperitoneal hematoma is generally caused in association with trauma or iatrogenic injury.^[7]^ Guha and Poole reported a case of hematoma under iliacus fascia on the tenth postoperative day after harvesting the anterior iliac crest bone for a bone graft.^[8]^ In addition, Nakamura et al reported an iliacus muscle hematoma case 6 months after revision hip arthroplasty.^[9]^ Although there are relatively rare cases of spontaneous retroperitoneal hematoma that are not associated with trauma or iatrogenic injury, they can occur due to coagulopathies, vascular lesions, or pathology.^[7]^ The incidences of retroperitoneal hematoma related to hemophilia or therapeutic anticoagulation were reported to be 10.4% in hemophilia patients and from 1.3% to 6.6% in those who use anticoagulants.^[4]^

Anticoagulant therapy, one of the most widely used medical therapies, is applied to cases receiving treatments for acute venous thromboembolism, atrial fibrillation, acute coronary syndrome, and invasive cardiac procedures, and is the core for treatment and prevention of thrombosis in various clinical settings.^[1]^ However, as the indication and use of anticoagulant therapy increase, the resulting anticoagulant associated complications are also increasing.^[6]^ Bleeding is the major complication of anticoagulant therapy and also a potential risk factor for all anticoagulants even when it is maintained within the usual therapeutic range.^[1]^

The patient reported in this case had no underlying diseases and no history of anticoagulation drug use. We performed emergency trauma management of the upper arm, including treatment for the accompanying medical problems and anticoagulant therapy for venous thrombosis, for a patient with a crush injury of the upper arm. To monitor if intravenous heparin therapy was administered at the appropriate dose, we also maintained a therapeutic range through about 4 sessions a day of activated partial thromboplastin time test. However, a large number of bilateral retroperitoneal hematomas occurred in the patient on the 5th day of anticoagulant therapy. The retroperitoneal hematomas were life threatening and extended from the right retroperitoneum to the femoral canal along the iliacus muscle. Since the fascia covering the iliacus muscle and femoral nerves are not easily stretchable,^[10]^ the hematoma in this region caused compression on the femoral nerve. In addition, rigid inguinal ligament induced further femoral nerve compression, and continuous compression on the femoral nerve resulted in femoral neuropathy. Goodfellow et al also reported that fluid injection into the iliacus muscle or the fascial sheath caused high pressure, compressing the femoral nerve against psoas muscle tendon, inducing ischemic femoral neuropathy.^[11]^

Although there are several reports of compressive femoral neuropathy induced by retroperitoneal hematoma as a complication of anticoagulant therapy, it is substantially rare.^[12]^ In a case series, Parmer et al reported cases of femoral nerve palsy due to spontaneous retroperitoneal hematoma in patients with chronic lymphocytic leukemia who received warfarin for recurrent pulmonary emboli and patients who received heparin postoperatively for atrial fibrillation after receiving mitral valve replacement.^[13]^ Previous case reports were on spontaneous retroperitoneal hematoma in patients who had continuous anticoagulant therapy for underlying diseases. In contrast, the present case is the first case that had bilateral spontaneous retroperitoneal hematomas caused by heparin, which was the first administered for the upper arm trauma management in a patient without underlying disease, and then was accompanied by compressive femoral neuropathy.

The diagnosis of retroperitoneal hematoma can be performed safely and simply through CT, and CT plays an important role in accurately determining its degree and location.^[14]^ However, it is difficult for CT to reveal injuries in the femoral nerve caused by compression. In this case, NCS and needle EMG can help diagnose femoral neuropathy.^[15]^ We could not identify nerve conduction in a motor NCS for the femoral nerve and a sensory NCS for the saphenous nerve. In addition, needle EMG identified denervation potentials in the motor branches of the femoral nerve, including iliopsoas, rectus femoris, and vastus medialis muscles. Finally, a CT examination found a large number of bilateral retroperitoneal hematomas that extended along the femoral canal, confirming that the patient had damage in the femoral nerve caused by compression.

Since spontaneous retroperitoneal hematomas causing femoral neuropathy are rare, its treatment is also controversial.^[^2^,^^4^^]^ While conservative treatment was reported beneficial to hematological pathology, emergency surgical intervention was suggested for progressive neuropathy, particularly when there is no disorder of hemostasis.^[2]^ In the present case, conservative treatment was performed together with close neurological and hemodynamic monitoring after emergency embolization for the lumbar artery with active bleeding. The patient showed rapid improvements in vital signs, pain, and motor weakness after early vascular intervention and comprehensive physical therapy, which would result in a desirable prognosis.

## 4 Conclusion

Spontaneous retroperitoneal hematoma is a complication that is rare but can occur during anticoagulant therapy. It should be suspected if a patient shows abdominal distension, anemia, and hemodynamic instability during anticoagulant therapy. Even anticoagulant therapy for emergency care can cause retroperitoneal hematomas. Retroperitoneal hematomas can be accompanied by compressive femoral neuropathy that may induce muscle weakness in the proximal lower extremity and groin pain. A rapid diagnosis through CT and electrodiagnostic study and appropriate treatment planning would positively affect prognosis.

## Author contributions

**Conceptualization, literature review, manuscript revision:** Hwan-Kwon Do.

**Literature review, general supervision:** Da-Jung Lee.

**Anatomical review, linguistic supervision:** Wanil Kim.

**Literature review, writing of manuscript:** Tae-Hoon Kim.

**Data curation:** Tae-Hoon Kim, Da-Jung Lee.

**Formal analysis:** Wanil Kim.

**Supervision:** Da-Jung Lee, Wanil Kim, Hwan-Kwon Do.

**Writing – original draft:** Tae-Hoon Kim.

**Writing – review & editing:** Hwan-Kwon Do.
